# Scarce quality assurance documentation in major clinical trial registries for approved medicines used in post-marketing clinical trials

**DOI:** 10.1186/s13063-019-3277-8

**Published:** 2019-04-11

**Authors:** Yorokpa Joachim Doua, Hanneke Dominicus, Julius Mugwagwa, Suzelle Magalie Gombe, Jude Nwokike

**Affiliations:** 1 Consortium for African Regulatory Expertise Development (CARED), Voorburg, The Netherlands; 2 Clinical and Medical Department, Janssen Infectious Diseases and Vaccines, Crucell Holland BV, Leiden, The Netherlands; 3 Dominicus Medicus Consultancy, Voorburg, The Netherlands; 4 Department of Science, Technology, Engineering and Public Policy, University College London, London, UK; 5 Exphar pharma, Thines, Belgium; 6 United States Pharmacopeial Convention (USP), Promoting the Quality of Medicines (PQM) Program, Washington, DC USA

**Keywords:** Clinical trial, drug registry, investigational medicinal product, drug regulation, product quality

## Abstract

**Background:**

This research reviewed major Clinical Trial Registries (CTRs) and assessed the availability of fields on quality assurance for approved medicines used as Investigational Medicinal Products (IMPs) in phase IV clinical trials.

**Methods:**

Two reviewers independently assessed CTRs of the International Committee of Medical Journal Editors (ICJME) and of World Health Organization (WHO) platforms. Each CTR was checked by two reviewers on availability of fields on brand name, manufacturer’s name, approval status, approving authority, compliance with Good Manufacturing Practices, and quality testing. In case of discrepancy, consensus was sought between the two reviewers.

**Results:**

Of 19 identified CTRs, 8 and 6 belonged to WHO and ICMJE, respectively, while 5 were equally part of both platforms. All CTRs had an ‘intervention’ field where data on IMPs and IMP comparators are captured. The Canadian CTR used ‘drug name’ rather than ‘intervention’. The EU, Peruvian, and UK CTRs had fields for ‘brand name’. However, only the EU CTR had fields for ‘manufacturer’s name’, ‘approval status’, and ‘approving authority’. None of the CTRs had fields on ‘compliance with Good Manufacturing Practices’ or ‘quality testing’.

**Conclusion:**

This study demonstrates that none of the CTRs of ICMJE and WHO platforms has adequate fields to establish that the source of post-marketing IMPs is of assured quality. This is astonishing given the lengthy requirements in WHO and ICMJE guidelines. Considering the relation between IMP quality and safety of clinical trial participants, the gap of quality assurance fields should be bridged at CTRs concurrently to adjustments of WHO and ICMJE guidelines on CTRs. Specifically, IMP quality testing addressing issues on IMP appearance, impurities, microbial contamination, and dosing should be conducted and reported before, during, and after clinical trial conduct. Until adoption of these measures, the EU CTR should be preferred for registration of phase IV clinical trials conducted in countries lacking stringent regulatory capacities.

**Electronic supplementary material:**

The online version of this article (10.1186/s13063-019-3277-8) contains supplementary material, which is available to authorized users.

## Background

Randomized clinical trials (RCTs) are the gold standard among research designs for assessing the efficacy and safety of medicines before they enter the market; they lead the therapeutic choice in healthcare [1]. The strength of RCTs lies in their ability to control for confounders like differences among patient baseline characteristics, concurrent diseases, and concurrent treatments that may distort the effect of the interventions under investigation [2]. The randomization of RCTs equally distributes potential confounders between the study groups, making the study medications the unique difference. Therefore, the therapeutic effect that is observed between the treatment group and the control group is attributed to the investigational medicinal product (IMP). An IMP may be a new chemical entity in development when used in phase I–III clinical trials, and it can also be an approved medicine that is further investigated to assess its effect in real-life when used in phase IV clinical trials.

To deliver the therapeutic effect, avoid drug resistance and harm to patients, and to ensure reliability of the clinial study results, the IMP must comply with quality standards [3–5]. Unfortunately, measures to comply with these quality standards seem to be lacking in non-commercial clinical trial protocols [6].

Medicines of poor quality are Substandard and Falsified (SF) medicines that are described by three terms. Firstly, substandard medicines, also called out-of-specification, are authorized medical products that fail to meet either their quality standards or specifications, or both. Secondly, unregistered or unlicensed medicines are those that have not undergone evaluation and approval by the National or Regional Regulatory Authority for the market in which they are marketed or used. Lastly, falsified medicines, which deliberately or fraudulently misrepresent their identity, composition or source [7]. All three types of SF medicines are assessed by drug regulatory authorities (DRAs) based on criteria determining their quality (Table 1). Falsified medicines especially will need involvement of law and security enforcement authorities. Unfortunately, that assessment requires sophisticated equipment and reagents and high technical abilities which most DRAs in low- and middle-income countries (LMICs) cannot afford [8]. Research on the quality of marketed medicines has shown worryingly high rates of SF medicines in LMICs [9–14]. In some African countries, for instance, substandard antimalarials were associated to 122,000 deaths of children under-five years [7].

**Table 1:** Factors determining quality of medicines

Active pharmaceutical ingredients	
Specifications of Finished Products	
Stability	
Therapeutic equivalence (to the innovator)	
Packaging, labelling, product information	
Good Distribution Practices, Good Storage Practices	
Dosing	
Bio-availability	
Impurities (e.g., diethylene glycol)	
Decreased efficacy of the active ingredient	
Cross-contamination with highly active molecules	
Sterility	
Microbiological contamination	

While regulatory-approved medicines of poor quality may be used as IMPs in post-marketing clinical trials, attention has not been sufficiently given to the quality of approved IMPs as they were considered exempt from quality compliance issues until publications of product quality defect reports in clinical trials in 2016 [15]. One of these reports described vitamin A capsules, manufactured in Canada and Italy and supplied by WHO, which were used in a clinical trial in Tanzanian infants; owing to quality checks, the product was proved degraded by 32% toward the clinical trial completion [16]. Another report indicates that clopidogrel of brand Plavix, which prevents heart attacks and strokes, could not be used in the US as an IMP comparator in 2007 after quality testing detected only 50–80% of the active ingredient in the product [17]. These two facts suggest that approved IMPs should not be exempted from quality compliance issues.

The Good Clinical Practice guidelines make clinical trial sponsors accountable for IMP quality [18, 19]. The same guidelines require clinical trial sponsors to describe quality assurance measures of IMPs in a clinical trial protocol, which must be registered in a Clinical Trial Registry (CTR) for approval [19]. While commercial study sponsors might be used to the quality assurance requirements of an IMP, non-commercial sponsor-investigators may not [6, 20]. Yet, non-commercial clinical trials are essential for the development and the post-marketing monitoring of medicines of global public health interest.

In this paper, we present an assessment of documentations in major CTRs regarding product quality, argue for assessment of the quality of approved IMPs and propose recommendations for enhancing relevant regulation updates.

## Methods

For the assessment of the CTRs, a checklist of eight items as highlighted in Table 2, was developed using criteria that determine a pharmaceutical product’s quality. The most common quality attributes of IMPs are the appearance, impurities, microbial contamination, and dosing. However, other items, namely the product brand name, manufacturer’s name, and regulatory approval status, were added to that checklist as they may impact product quality. The CTRs of interest in this study were those fulfilling international guidelines. CTRs of the WHO International Clinical Trial Registries platform (WHO-ICTRP) or those recommended by the International Committee of Medical Journals Editors (ICMJE) were targeted [21, 22]. Only the English versions of these CTRs were screened. For each CTR, the data fields were viewed by displaying a registered study protocol owing to the search functionality. ‘Phase IV clinical trial’ was used as the search term, entered in the search window and a search was subsequently launched to display a list of registered study protocols. One of the displayed study protocols was then opened randomly and the product quality-related data fields were assessed. The data was further collected by a reviewer in line with the checklist and entered in a spreadsheet for analysis. To ensure accuracy, the collected data was reassessed by a second reviewer. In case of discrepancies, a consensus was sought between the two reviewers.

**Table 2:** Clinical Trial Registry checklist for product quality

Items	
Intervention or active pharmaceutical ingredient	
Brand	
Manufacturer’s name	
Good manufacturing practice certificate	
Good manufacturing practice site	
Quality control testing	
Drug regulatory approval	
Approving regulatory authority	

## Results

IMP quality-related data of CTRs are displayed in Table 3. Nineteen CTRs meeting the selection criteria were screened to assess the availability of quality-related data for IMPs; eight and six of these CTRs belonged to the WHO-ICTRP and ICMJE platforms, respectively, while the remaining five were equally part of both platforms (these were the CTRs of the European Union,

**Table 3:** Quality-related data field for IMPs in clinical trial registry of ICJME^a^ and WHO-ICTRP^b^

Source registry	Intervention or active pharmaceutical ingredient (Y/N)	Brand (Y/N)	Manufacturer’s name (Y/N)	Good Manufacturing Practice certificate (Y/N)	Good Manufacturing Practice site (Y/N)	Quality control testing (Y/N)	Drug regulatory approval (Y/N)	If yes, approving regulatory authority (Y/N)
Australian New Zealand Clinical Trials Registry (ANZCTR)^c^	Y	N	N	N	N	N	N	N
Brazilian Clinical Trials Registry (ReBec)^c,d^	Y	N	N	N	N	N	N	N
Chinese Clinical Trial Registry (ChiCTR)^c,d^	Y	N	N	N	N	N	N	N
Clinical Research Information Service Republic of Korea (CRiS)^c,d^	Y	N	N	N	N	N	N	N
Clinical Trials Registry – India (CTRI)^c^	Y	N	N	N	N	N	N	N
Cuban Public Registry of Clinical Trials (RPCEC)^c^	Y	N	N	N	N	N	N	N
EU Clinical Trials Register (EU-CTR)^c,d^	Y	Y	Y	N	N	N	Y	Y
German Clinical Trials Register (DRKS)^d^	Y	N	N	N	N	N	N	N
Iranian Registry of Clinical Trials (IRCT)^c^	Y	N	N	N	N	N	N	N
ISRCTN^c^	Y	Y	N	N	N	N	N	N
Japan Primary Registries Network (JPRN)^c,d^	Y	N	N	N	N	N	N	N
Thai Clinical Trials Registry (TCTR)^d^	Y	N	N	N	N	N	N	N
The Netherlands National Trial Register (NTR)^d^	Y	N	N	N	N	N	N	N
Pan African Clinical Trials Registry (PACTR)^c^	Y	N	N	N	N	N	N	N
Peruvian Clinical Trial Registry (REPEC)^c^	Y	Y	N	N	N	N	N	N
Sri Lanka Clinical Trials Registry (SLCTR)^c^	Y	N	N	N	N	N	N	N
Clinicaltrials.gov^d^	Y	N	N	N	N	N	N	N
Swiss National Clinical Trials Portal (SNCTP)^d^	Y	N	N	N	N	N	N	N
Health Canada Clinical Trials Database^d^	Y	N	N	N	N	N	N	N

^a^ICMJE stands for International Committee for Medical Journals Editors

^b^WHO-ICTRP stands for WHO International Clinical Trial Registry Platform

^c^Clinical Trial registry of ICTRP

^d^ICMJE-recommended clinical trial registries

Japan, Brazil, China, and of the Korean Republic). All CTRs had a search functionality enabling the display of the registered clinical trial protocols.

All CTRs had an ‘intervention’ data field where data on the IMPs and the IMP comparators are captured. Health Canada’s CTR used ‘drug name’ rather than ‘intervention’. Three CTRs, namely the European, Peruvian, and that of United Kingdom’s BioMed Central, had a data field for ‘brand name’. Only the European CTR had data fields for ‘manufacturer name’, ‘product approval status’, and ‘approving DRA’. Of all CTRs that were screened, neither data fields were available on ‘compliance with good manufacturing practices’ (cGMP) nor on ‘product quality testing’.

## Discussion

This study demonstrates that none of the CTRs in the WHO-ICTRP and ICMJE platforms have sufficient data fields to establish that the source of IMPs used in phase IV clinical trials is of assured quality. These findings are astonishing since they contrast the lengthy quality requirements outlined in WHO as well as International Conference on Harmonisation guidelines [4, 5].

Three patterns, namely the product’s regulatory approval status, cGMP, and quality testing, directly impact product quality. A regulatory approval is evidenced either by a listing of medicinal products in a regulatory approval database or a certificate of pharmaceutical product. It shows the legal source of a pharmaceutical product. However, proving the legal source of an IMP based on listing in a regulatory database may be a challenge in regions of the globe such as Africa, where only few regulatory databases are available [23]. A certificate of pharmaceutical product is therefore a valuable proof of registration. Moreover, it is relevant to have information on the approving regulatory authority since only regulatory approvals provided by a stringent DRA have high quality standards [24]. Unfortunately, as it can be seen from the results, data fields on the regulatory approval status of the IMP as well as the approving DRAs are lacking in most major CTRs except the European CTR. That gap should therefore be bridged by adding proof of registration and the approving authority in CTRs to prevent use of falsified IMPs by clinical trial participants.

When the regulatory approval status is unspecified, the brand and the manufacturer’s name may serve as starting points towards investigating whether an IMP is approved or falsified. However, as shown by the results, only a few CTRs have data fields on brand and manufacturer name. Brand and manufacturer name alone, including those of global companies, cannot guarantee quality assurance of approved IMPs since reports exist on quality defects of US-approved products of trusted and reputable global manufacturers [15, 25]. When combined with a brand and manufacturer name, the regulatory approval is a better indicator of the IMP legal source [5]. Nevertheless, the results of this study showed a data gap on brand and manufacturer name, which must be included in CTRs.

A regulatory approval suggests that the approving DRA acknowledges the manufacturer’s cGMP. However, like the regulatory approval status, only a certificate of cGMP provided by a stringent DRA assures the quality of approved IMPs used in LMICs in line with the reported regulatory weaknesses in these countries. A cGMP certificate and quality testing provide the highest quality assurance for approved IMPs.

Indeed, a cGMP certificate sustains the quality standards of the manufacturing process and allows identification of substandard medicines. In turn, quality testing provides information on the integrity of the active ingredient and equally assures a conformant dosing. Thus, when the analytical method is valid, a quality testing enables the identification of product degradation. Nevertheless, none of the CTRs had data fields on cGMP and quality testing, as shown by the study results. Therefore, the gap of data on cGMP and quality testing at CTRs should also be bridged to prevent the use of substandard and degraded IMPs in phase IV clinical trials.

## Conclusion

Currently, none of the major CTRs has sufficient data fields on quality assurance of an approved IMP and consequently do not promote the quality of IMPs in post-marketing phase IV clinical trials. Considering the potential impact of product quality on the safety and wellbeing of clinical trial participants but also on the validity of clinical trial results, these gaps represent a concern for public health. This warrants that data fields on product brand name, manufacturer name, cGMP, regulatory approval status, and quality testing be added to CTRs. It is especially relevant that prior quality testing be conducted for IMPs before, during, and at completion of non-commercial clinical trials and sponsor-investigators should specifically report on these testing results. To this end, WHO international standards on CTRs and the ICMJE clinical trial registration policy should be adjusted. However, for an efficient implementation of these guidelines, a concurrent adjustment of WHO and International Conference on Harmonisation clinical trial regulations and the CONSORT guidelines is also necessary, as previously suggested [15]. Nevertheless, until these are completed and the identified gaps in product quality assurance data are bridged in CTRs, the European CTR should be preferred for post-marketing non-commercial clinical trials conducted in LMICs lacking stringent DRAs (Additional file 1).

## Additional files

Additional file 1: Reporting specific parameters to increase reproducibility of database studies. (DOCX 20 kb)
