# Supplementary material for: Scarce quality assurance documentation in major clinical trial registries for approved medicines used in post-marketing clinical trials
Source: Trials. 2019 Apr 11;20:212. doi: 10.1186/s13063-019-3277-8 (PMC6460528; doi:10.1186/s13063-019-3277-8)
Supplement: Supplementary file 1 — Reporting specific parameters to increase reproducibility of database studies. (DOCX 20 kb) [file 13063_2019_3277_MOESM1_ESM.docx]

**Reporting to Improve Reproducibility and Facilitate Validity Assessment for Healthcare Database Studies V1.0**

**Additional file 1 – Reporting specific parameters to increase reproducibility of database studies*.**

| **Items** | **Description** | **Information** |
| --- | --- | --- |
| **A. Reporting on data source should include:** | | |
| A.1 Data provider | Data source name and name of organization that provided data. | Data was collected by the reviewers from the following registers:   - Australian New Zealand Clinical Trials Registry (ANZCTR) - Brazilian Clinical Trials Registry (ReBec) - Chinese Clinical Trial Registry (ChiCTR) - Clinical Research Information Service Republic of Korea (CRiS) - Clinical Trials Registry - India (CTRI) - Cuban Public Registry of Clinical Trials(RPCEC) - EU Clinical Trials Register (EU-CTR) - German Clinical Trials Register (DRKS) - Iranian Registry of Clinical Trials (IRCT) - ISRCTN - Japan Primary Registries Network (JPRN) - Thai Clinical Trials Registry (TCTR) - The Netherlands National Trial Register (NTR) - Pan African Clinical Trials Registry (PACTR) - Peruvian Clinical Trial Registry (REPEC) - Sri Lanka Clinical Trials Registry (SLCTR) - Clinicaltrials.gov - Swiss National Clinical Trials Portal (SNCTP) - Health Canada Clinical Trials Database |
| A.2 Data extraction date (DED) | The date (or version number) when data were extracted from the dynamic raw transactional data stream (e.g. date that the data were cut for research use by the vendor). | 23-August-2017 |
| A.3 Data sampling | The search/extraction criteria applied if the source data accessible to the researcher is a subset of the data available from the vendor. | “Phase IV clinical trial” |
| A.4 Source data range (SDR) | The calendar time range of data used for the study. Note that the implemented study may use only a subset of the available data. | Not applicable |
| A.5 Type of data | The domains of information available in the source data, e.g. administrative, electronic health records, inpatient versus outpatient capture, primary vs secondary care, pharmacy, lab, registry. | Clinical trials registries |
| A.6 Data linkage | other supplemental data Data linkage or supplemental data such as chart reviews or survey data not typically available with license for health care database. | No |
| A.7 Data cleaning | Transformations to the data fields to handle missing, out of range values or logical inconsistencies. This may be at the data source level or the decisions can be made on a project specific basis. | No |
| A.8 Data model | conversion Format of the data, including description of decisions used to convert data to fit a Common Data Model (CDM). | None |
| **B. Reporting on overall design should include:** |  |  |
| **C. Reporting on inclusion/exclusion criteria should include:** | | |
| C.1 Study entry date (SED) | The date(s) when subjects enter the cohort. | Not applicable |
| C.2 Person or episode level study entry | The type of entry to the cohort. For example, at the individual level (1x entry only) or at the episode level (multiple entries, each time inclusion/ exclusion criteria met). | Not applicable |
| C.3 Sequencing of exclusions | The order in which exclusion criteria are applied, specifically whether they are applied before or after the selection of the SED(s). | Not applicable |
| C.4 Enrollment window (EW) | The time window prior to SED in which an individual was required to be contributing to the data source. | Not applicable |
| C.5 Enrollment gap | The algorithm for evaluating enrollment prior to SED including whether gaps were allowed. | Not applicable |
| C.6 Inclusion/ Exclusion definition window | The time window(s) over which inclusion/exclusion criteria are defined. | Not applicable |
| C.7 Codes | The exact drug, diagnosis, procedure, lab or other codes used to define inclusion/exclusion criteria. | Not applicable |
| C.8 Frequency and temporality of codes | The temporal relation of codes in relation to each other as well as the SED. When defining temporality, be clear whether or not the SED is included in assessment windows (e.g. occurred on the sameday,2codesforAoccurredwithin7days of eachotherduringthe30dayspriortoand including the SED). | Not applicable |
| C.9 Diagnosis position (if relevant/available) | The restrictions on codes to certain positions, e. g. primary vs. secondary. Diagnoses. | Not applicable |
| C.10 Care setting | The restrictions on codes to those identified from certain settings, e.g. in patient, emergency department, nursing home. | Not applicable |
| C.11 Washout for exposure | The period used to assess whether exposure at the end of the period represents new exposure. | Not applicable |
| C.12 Washout for outcome | The period prior to SED or ED to assess whether an outcome is incident. | Not applicable |
| **D. Reporting on exposure definition** | - | Not applicable |
| **E. Reporting on follow‐up time** | - | Not applicable |
| **F. Reporting on outcome definition** | - | Not applicable |
| **G. Reporting on covariate definitions** | - | Not applicable |
| **H. Reporting on control sampling** | - | Not applicable |
| **I. Reporting on statistical software** | - | Not applicable |
